# The M6A methyltransferase METTL3: acting as a tumor suppressor in renal cell carcinoma

**DOI:** 10.18632/oncotarget.21726

**Published:** 2017-10-10

**Authors:** Xiao Li, Jingyuan Tang, Wen Huang, Feng Wang, Pu Li, Chao Qin, Zhiqiang Qin, Qing Zou, Jifu Wei, Lixin Hua, Haiwei Yang, Zengjun Wang

**Affiliations:** ^1^ State Key Laboratory of Reproductive Medicine, Department of Urology, First Affiliated Hospital of Nanjing Medical University, Nanjing 210029, China; ^2^ Department of Urology, Affiliated Cancer Hospital of Jiangsu Province of Nanjing Medical University, Nanjing 210009, China; ^3^ Department of Urology, Jiangsu Province Hospital of TCM, Affiliated Hospital of Nanjing University of TCM, Nanjing 210029,China; ^4^ Research Division of Clinical Pharmacology, First Affiliated Hospital of Nanjing Medical University, Nanjing 210029, China

**Keywords:** methyltransferase, METTL3, renal cell carcinoma

## Abstract

We aimed to study the role of METTL3 in renal cell carcinoma (RCC) carcinogenesis and development. Immunohistochemistry was performed in clinical tissue microarray. Expression level of METTL3 in RCC tissues and cell lines was evaluated by quantitative real-time PCR (qRT-PCR) and western blot. Then, the effects of METTL3 on proliferation, migration, invasion and cell cycle were studied in RCC cells. Additionally, *in vivo* study was carried out in nude mice. Negative METTL3 expression was associated with larger tumor size (P=0.010) and higher histological grade (P=0.021). Moreover, RCC patients with positive METTL3 expression had an obvious longer survival time (*P*=0.039). METTL3 mRNA and protein expression was lower in RCC samples compared with adjacent non-tumor samples, and lower in RCC cell lines (CAKI-1, CAKI-2 and ACHN) compared with HK-2. Afterwards, knockdown of METTL3 could obviously promote cell proliferation, migration and invasion function, and induce G0/G1 arrest. In contrast, up-regulation of METTL3 could inhibit such functions and reduce G0/G1 arrest. Additionally, up-regulation of METTL3 significantly suppressed tumor growth *in vivo*. Furthermore, significant changes in epithelial-to-mesenchymal transition (EMT) and PI3K-Akt-mTOR pathways were observed. Overall, our findings demonstrated that METTL3 might have a carcinostasis role in cell proliferation, migration, invasion function and cell cycle of RCC, indicating METTL3 may act as a novel marker for tumorigenesis, development and survival of RCC.

## INTRODUCTION

Renal cell carcinoma (RCC) is currently the 9th most common cancer in men and 14th most common in women worldwide [1]. The mortality of RCC has continuously increased by approximately 1.5-5.9% every year [2]. About 25% of patients are diagnosed with locally advanced or metastatic disease at first. However, 20-40% patients of local RCC with history of surgical resection will develop metastatic RCC subsequently [3, 4]. Metastasis occurs in the later stage and predicts very poor clinical outcome. Moreover, RCC is unique in that it is relatively resistant to radiotherapy and does not respond to standard chemotherapy [5, 6]. Though cytokine therapy and molecular targeting therapy have been applied for RCC, the therapeutic effects of these are limited with a short interval [7]. Now the overall survival of RCC remains poor. Thus, the detailed molecular mechanism underlying the RCC carcinogenesis requires to be fully clarified [8].

It is emerging that N6-methyladenosine (m^6^A) is a conserved internal modification in almost all eukaryotic nuclear RNAs [9, 10], playing important and diverse roles in numerous biological processes [11–13]. This reversible modification is catalyzed by m^6^A demethylation and methylation. METTL3 was originally identified as a methyltransferase responsible for m^6^A modification. It is known as S-adenosylmethionine (SAM)-binding subunit associated with mRNA methylation, which acts as one part of the methyltransferase complex [14, 15]. Although m^6^A methyltransferase activity has been detected in both nuclear and cytoplasmic extracts, METTL3 (as well as the other complex components) are observed predominantly in nuclear speckles by immunofluorescence analyses.

Some studies found that METTL3 regulated translation of specific mRNAs containing m^6^A peaks near the stop codon. Furthermore, METTL3 could bind to other regions of mRNA, such as 5’ CDS region, to promote translation. It is reported that METTL3 promotes growth, survival, and invasion of human lung cancer cells by enhancing mRNA translation through an interaction with the translation initiation machinery [15, 16]. Studies also revealed METTL3 appears to be strongly associated with promoting translation of oncogenesis in cancer. Accordingly, exploring the functional role and underlying molecular mechanism of METTL3is necessary.

In this study, we first analyzed METTL3 expression in clinical RCC tissue with immunohistochemistry and survival analysis. Next, the potential biomedical function and mechanism of METTL3 were studied. Our results indicated that METTL3 may act as a novel diagnosis and prognostic biomarker for RCC patients.

## RESULTS

### Relationship between METTL3 expression and clinicopathological factors in RCC patients

**Figure 1:**
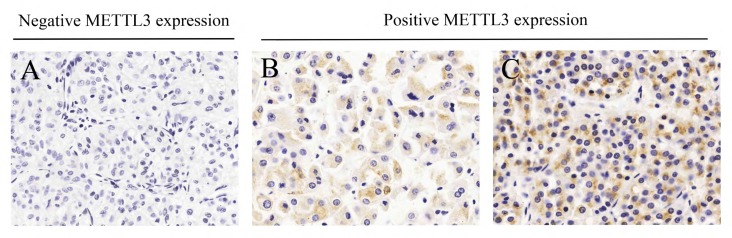
Different immunohistochemistry results of METTL3 expression in microarray **(A)** Negative METTL3 expression. **(B)** Weak METTL3 expression. **(C)** Strong METTL3 expression.

To assess the potential connection between METTL3 expression and clinicopathological factors in RCC patients, immunohistochemistry was performed in clinical tissue microarray (Figure 1). A total of 145 cases were included with comprehensive data of basic information, clinicopathology and survival (Supplementary Table 2). According to the immunohistochemistry results, 145 RCC patients were classified into two groups of positive METTL3 expression group (n=28) and negative METTL3 expression group (n=117). Clinicopathological factors were compared between these two groups (Table 1), demonstrating that negative METTL3 expression was associated with larger tumor size (P=0.010) and higher histological grade (P=0.021). However, METTL3 expression level was not associated with other parameters such as age, gender or tumor stage.

**Table 1:** Relationship of METTL3 expression and clinicopathologic characteristics of patients

Variable	Total (%)	METTL3 expression		
		Negative (n=117)	Positive (n=28)	*P* value
Age				0.197
<60	88	74 (84.3)	14 (15.7)	
≥60	57	43 (75.0)	14 (25.0)	
Gender				0.433
Male	89	70 (78.7)	19 (21.3)	
Female	56	47 (83.9)	9 (16.1)	
Tumor size				**0.010**
≤4	72	52 (69.1)	20 (30.9)	
>4	73	65 (86.4)	8 (13.6)	
Histology				**0.002**
Clear cell carcinoma	127	108 (85.0)	19 (15.0)	
Others	18	9 (50.0)	9 (50.0)	
Histological grade				**0.021**
I-II	107	79 (76.0)	25 (24.0)	
III-IV	38	38 (92.7)	3 (7.3)	
Tumor stage				0.090
T1	106	82	24	
T2-T4	39	35	4	

Bold: *P* < 0.05. *P* values were two-tailed and based on the Pearson chi-square test.

### Association of METTL3 expression and survival

**Figure 2:**
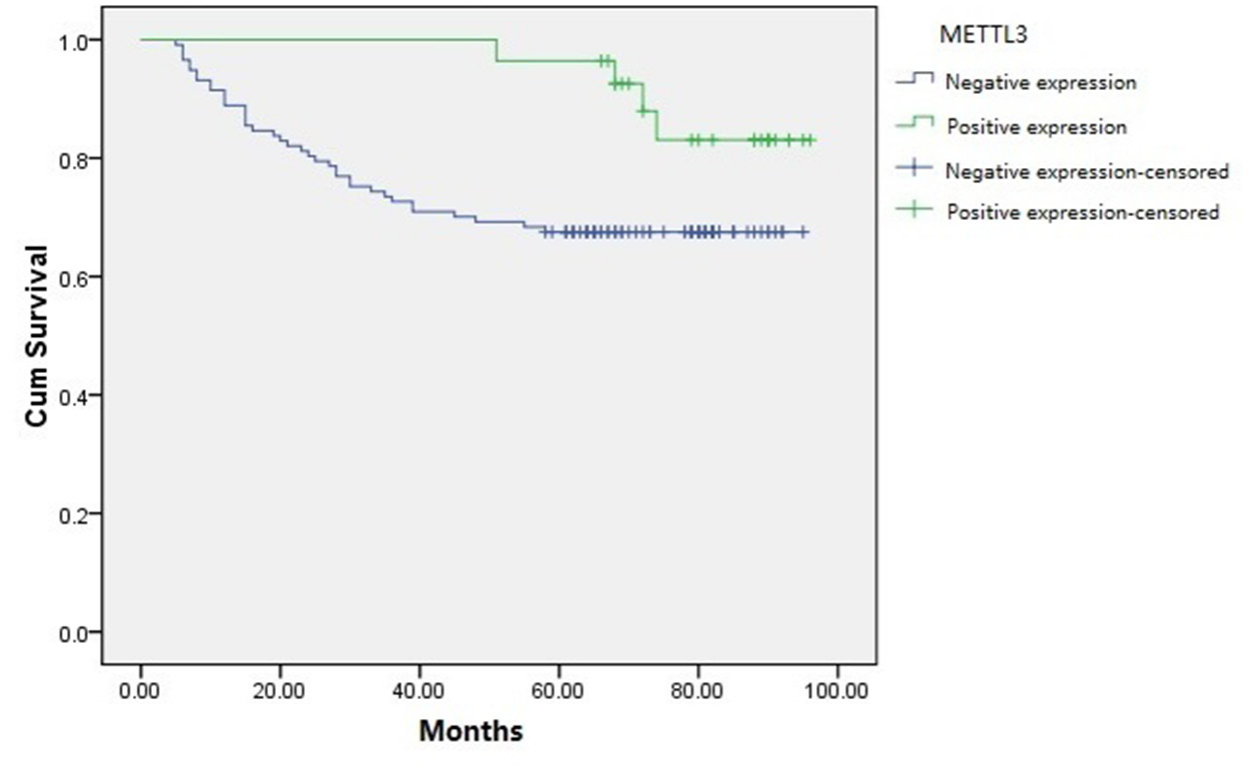
Kaplan-Meier survival curves of RCC patients based on METTL3 expression levels Patients with positive METTL3 expression had obviously longer survival time than those with negative METTL3 expression (log-rank test, *P*=0.039).

We further analyzed the correlation of METTL3 expression and survival outcome of RCC patients after partial or radical nephrectomy. Kaplan-Meier analysis (Figure 2) results showed that RCC patients with positive METTL3 expression had significantly longer survival time (log-rank test, *P*=0.039). As shown in Table 2, in univariate analysis, the survival of RCC patients was significantly associated with age (*P*=0.005), tumor size (*P*=0.015), histological grade (*P*=0.000), tumor stage (*P*=0.000) and METTL3 expression (*P*=0.049). Moreover, multivariate analysis indicated that age (*P*=0.001), histological grade (*P*=0.011), tumor stage (*P*=0.000) and METTL3 expression (*P*=0.026) were independent prognostic indexes for survival.

**Table 2:** Univariate and multivariate analyses for survival (Cox proportional hazards regression model)

Variable	Univariate analysis			Multivariate analysis		
	HR	95% CI	P	HR	95% CI	P
Age	2.419	1.312-4.459	**0.005**	3.057	1.618-5.774	**0.001**
≥60 vs. <60						
Gender	1.034	0.555-1.929	0.915			
Male vs. Female						
Smoking status	0.881	0.468-1.656	0.693			
Ever vs. Never						
Drinking status	0.833	0.409-1.694	0.613			
Ever vs. Never						
Hypertension	0.642	0.338-1.219	0.176			
Ever vs. Never						
Diabetes	1.091	0.505-2.359	0.824			
Ever vs. Never						
Tumor size	2.222	1.169-4.223	**0.015**			
<4 vs. ≤4						
Histology	1.339	0.478-3.753	0.579			
Clear cell carcinoma vs. Others						
Histological grade	4.603	2.492-8.503	**0.000**	2.486	1.235-5.004	**0.011**
III-IV vs. I-II						
Tumor stage	5.890	3.165-10.961	**0.000**	3.550	1.760-7.163	**0.000**
T2- T4 vs. T1						
METTL3 expression	0.355	0.126-0.994	**0.049**	0.300	0.104-0.866	**0.026**
Positive vs. Negative						

### Expression of METTL3 in renal cancer tissue samples and cell lines

**Figure 3:**
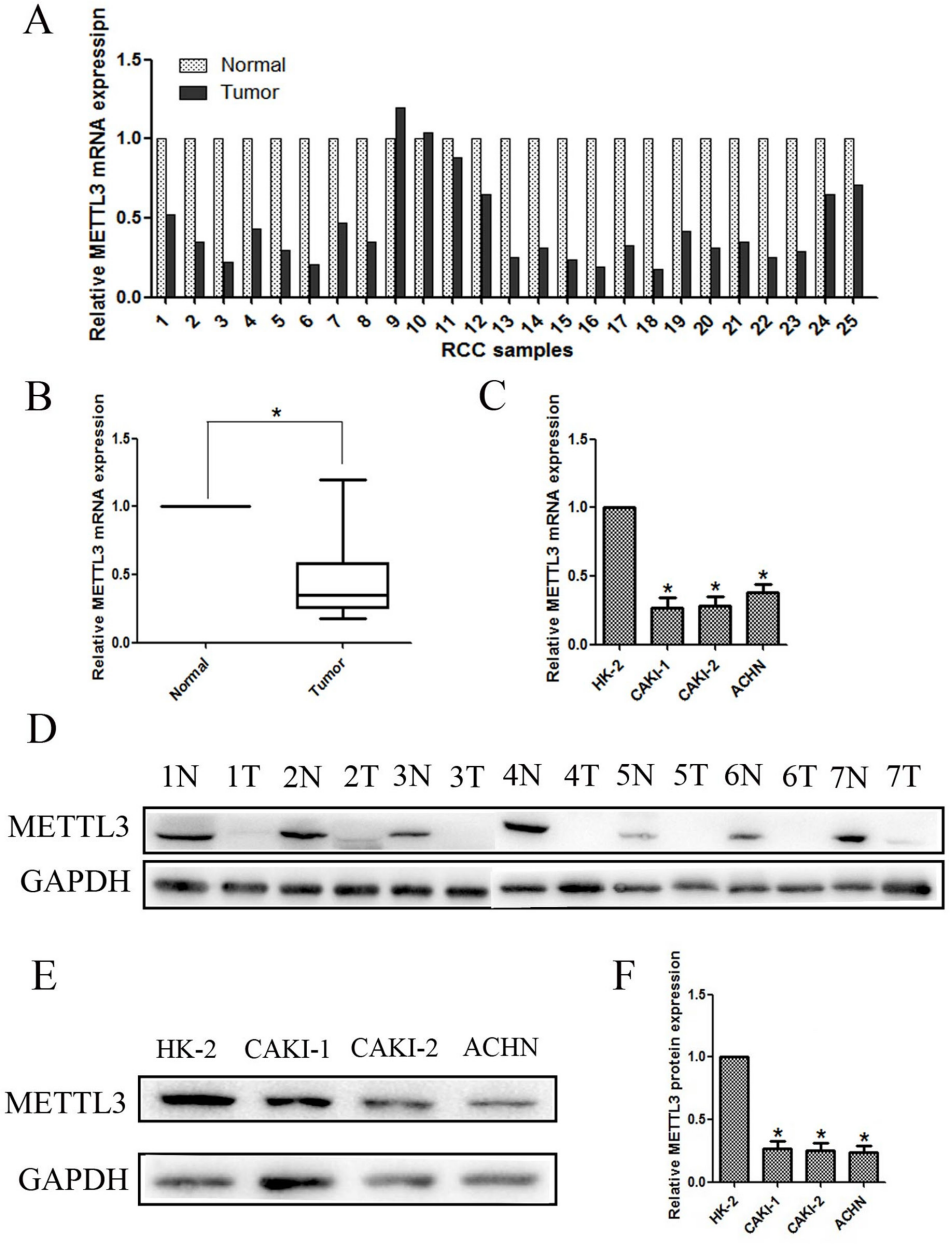
Expression of METTL3 in renal cancer tissue samples and cell lines **(A, B)** Relative mRNA expression of METTL3 in RCC tissues compared with corresponding non-tumor tissues. **(C)** Relative mRNA expression of METTL3 in RCC cell lines (CAKI-1, CAKI-2 and ACHN) and a normal human renal tubular epithelial cell line HK-2. **(D)** The expression of METTL3 in 7 randomly selected RCC samples compared with corresponding renal tissues. **(E, F)** Relative protein expression of METTL3 in RCC cell lines (CAKI-1, CAKI-2 and ACHN) and HK-2. ^*^P<0.05.

The mRNA expression of METTL3 was assessed using qRT-PCR in 25 human RCC samples and paired normal adjacent renal tissues. As shown in Figure 3AB, the results indicated that expression of METTL3 was significantly lower in RCC samples compared with adjacent non-tumor samples (*P*<0.05). We further examined METTL3 expression by qRT-PCR in three RCC cell lines (CAKI-1, CAKI-2 and ACHN) and a normal human renal tubular epithelial cell line HK-2, which demonstrated that METTL3 was also lower expressed in RCC cell lines than in HK-2 (Figure 3C).

Moreover, lower METTL3 expression was observed in 7 randomly selected RCC samples compared with corresponding renal tissues (Figure 3D). Additionally, lower METTL3 expression in protein level was also observed in RCC cell lines (CAKI-1, CAKI-2 and ACHN) compared with HK-2 (Figure 3EF). Overall, results demonstrated that METTL3 expression is lower in RCC, suggesting it might have influence on RCC tumorigenesis and development.

### Lentivirus-mediated knockdown and overexpression of METTL3

**Figure 4:**
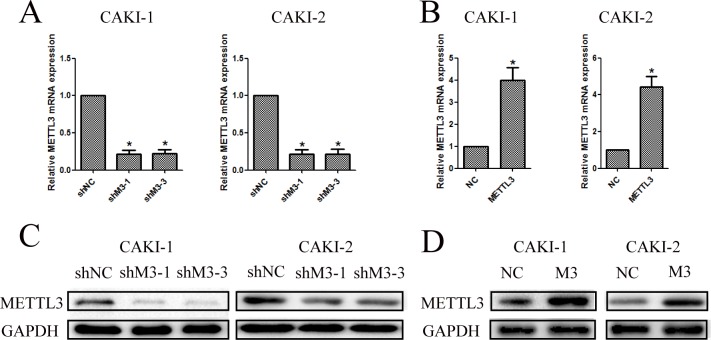
Knockdown and overexpression of METTL3 were confirmed **(A, B)** Knockdown and overexpression of METTL3 in CAKI-1 and CAKI-2 cell lines were confirmed with qRT-PCR to detect the relative mRNA expression of METTL3 (^*^P<0.05). **(C, D)** Knockdown and overexpression of METTL3 in CAKI-1 and CAKI-2 cell lines were confirmed with western blot to detect protein expression of METTL3 (^*^P<0.05).

To further investigate the function of METTL3 in RCC, we chose and infected CAKI-1 and CAKI-2 cells with lentivirus. Then, stably infected cell lines were selected for further study. The silenced cell lines were named as shMETTL3 (shM3), and the over-expressed cell lines were named as METTL3 (M3). The matched control cells were respectively named as shNC and NC in down-regulation and up-regulation groups. Three groups of RCC cell lines were silenced using different dose of lentivirus. While shM3-1 and shM3-3 had better transfection efficiency, which were thereby proceeded into subsequent experiments. The METTL3 expression levels were confirmed with qRT-PCR and western blot (Figure 4).

### METTL3 regulates cell proliferation

**Figure 5:**
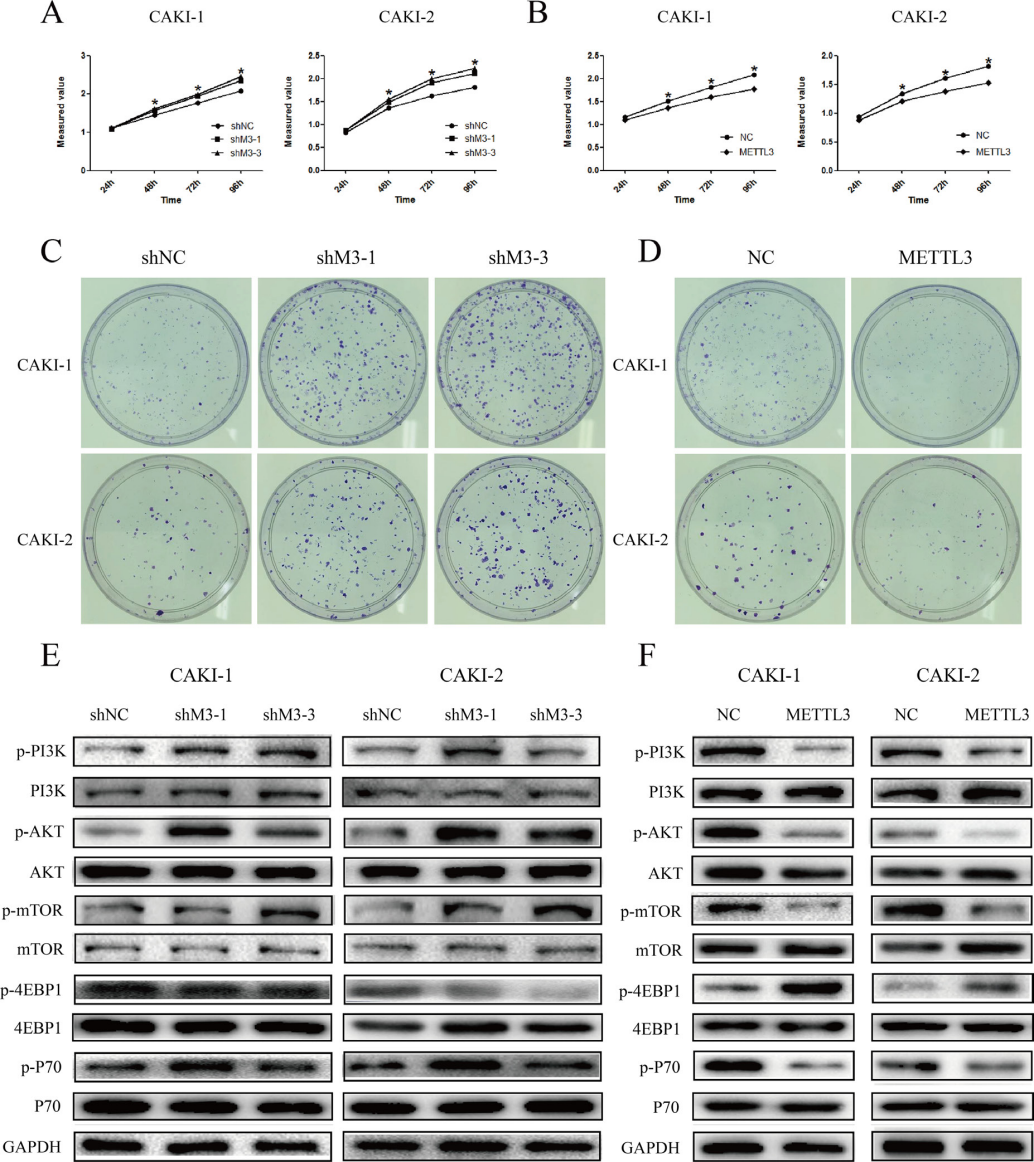
METTL3 regulates cell proliferation **(A, B)** Knockdown of METTL3 significantly promoted cell growth, while overexpression of METTL3 significantly inhibited cell growth. **(C, D)** Knockdown of METTL3 obvious increased the colony formation efficiency, while overexpression of METTL3 decreased the colony formation efficiency. **(E, F)** Knockdown of METTL3 promoted changes in pI3K/AKT/mTOR markers’ expression with a gain in p-PI3k, p-AKT, p-mTOR and p-p70, and a loss of p-4EBP1. While, overexpression of METTL3 promoted changes with a loss in p-PI3k, p-AKT, p-mTOR and p-p70, and a gain of p-4EBP1. GAPDH was used as a loading control. ^*^P<0.05.

CCK-8 assays were utilized to detect the impact of METTL3 on proliferation of RCC cells. The experiments revealed that down-regulation of METTL3 significantly promoted cell growth, while up-regulation of METTL3 significantly inhibited cell growth (*P*<0.05; Figure 5AB). Furthermore, the results of colony formation assay demonstrated that knockdown of METTL3 obvious increased the colony formation efficiency, yet overexpression of METTL3 decreased the colony formation efficiency (Figure 5CD).

Next, we investigated the effects of METTL3 on mTOR pathway. The results indicated that the expression of p-PI3k, p-AKT, p-mTOR, and p-P70 was obviously higher in sh-M3 groups, while the expression was lower in METTL3 overexpression group (Figure 5EF). Moreover, the expression of p-4EBP1 was significantly lower in sh-M3 groups, while the expression was higher in METTL3 overexpression group. However, no obvious changes were found in the expression of PI3k, AKT, mTOR, 4EBP1 and P70. These findings indicated that METTL3 might be involved in the promotion of RCC cell proliferation, and seems to be mediated by modulation of the PI3K-AKT-mTOR pathway.

### METTL3 regulates cellular migration and invasion

**Figure 6:**
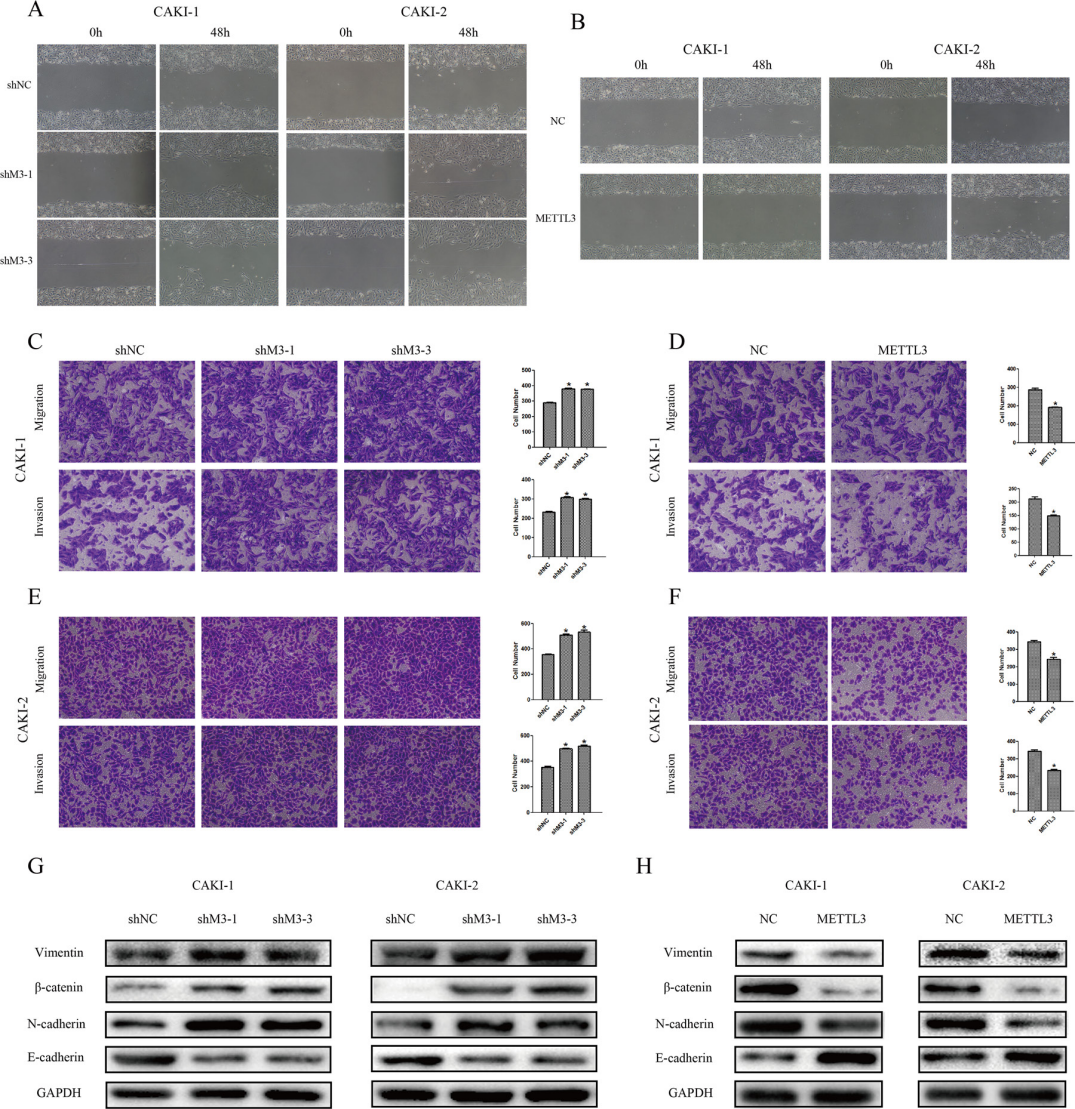
METTL3 regulates cellular migration and invasion **(A, B)** Knockdown of METTL3 promoted CAKI-1 and CAKI-2 cell wound-healing migration capacity, while overexpression of METTL3 inhibited wound-healing migration capacity. ^*^P<0.05. **(C-F)** Knockdown of METTL3 promoted cell migration and invasion capacities, while overexpression of METTL3 inhibited cell migration and invasion capacities. Original magnification 100×. ^*^P<0.05. **(G-H)** Knockdown of METTL3 promoted changes in EMT markers’ expression with a gain in vimentin, β-catenin and N-cadherin, and a loss of E-cadherin. While, overexpression of METTL3 promoted changes with a loss in vimentin, β-catenin and N-cadherin, and a gain of E-cadherin. GAPDH was used as a loading control. ^*^P<0.05.

Additionally, we explored the effect of METTL3 on RCC cell migration and invasion. First, we found that knockdown of METTL3 promoted cell wound-healing migration capacity, while overexpression of METTL3 inhibited the capacity (Figure 4AB). Moreover, the results showed that knockdown of METTL3 promoted transwell invasion capacity and migration capacity (p <0.05), while overexpression of METTL3 inhibited these capacities (Figure 6C-6F).

Accordingly to the results of cell functions in migration and invasion, we further detected relevant protein expression in epithelial-mesenchymal transition (EMT) pathway. The results revealed that the expressions of vimentin, β-catenin and N-cadherin were obviously higher and expression of E-cadherin was lower when METTL3 was down-regulated. Correspondingly, expressions of such proteins were in contrary when METTL3 was up-regulated (Figure 5GH), suggesting EMT pathway may be involved in the underlying mechanism.

### METTL3 regulates cell cycle

**Figure 7:**
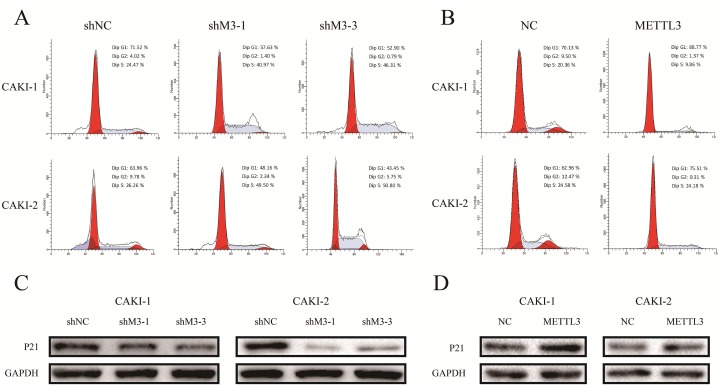
METTL3 regulates cell cycle **(A, B)** Down-regulation of METTL3 significantly decreased cell cycle arrest in G1 phase of cell cycle, whereas up-regulation of METTL3 increased cell cycle arrest in G1 phase. **(C, D)** Knockdown of METTL3 promoted a loss in P21 expression, while overexpression of METTL3 promoted a gain in P21 expression. GAPDH was used as a loading control. ^*^P<0.05.

Furthermore, flow cytometer was utilized to examine the effect of METTL3 on cell cycle progression. Down-regulation of METTL3 significantly decreased cell cycle arrest in G1 phase of cell cycle, whereas up-regulation of METTL3 increased cell cycle arrest in G1 phase (Figure 7AB). Moreover, knockdown of METTL3 promoted a loss in P21 expression, while overexpression of METTL3 promoted a gain in P21 expression (Figure 7CD).

### METTL3 significantly affected cellular growth *in vivo*

**Figure 8:**
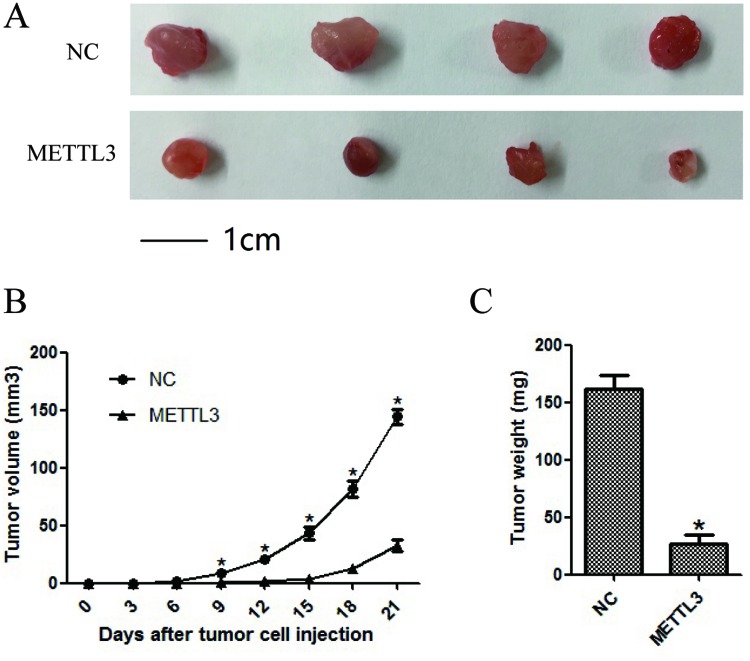
METTL3 significantly affected cellular growth *in vivo* **(A)** Representative pictures of tumors. **(B)** Tumor volumes were measured at the indicated number of days after mice were injected with tumor cells. **(C)** Final weight of tumors from each lentivirus treatment group. Each bar represented the mean tumor volume±S.D. or tumor weight±S.D. of four mice per group. ^*^P<0.05.

To investigate the effects of METTL3 expression on RCC cell growth *in vivo*, CAKI-1 cell line was chosen to assess tumorigenicity. Cell lines of NC and METTL3 were thus xenografted into nude mice. As shown in Figure 8A-8C, tumors derived from nude mice with overexpression of METTL3 grew obviously slower (all *P* <0.05).

## DISCUSSION

As the most common type of kidney cancer in adults, RCC accounts for 90-95% of neoplasms arising from the kidney [17]. Despite the development of therapeutic tool, the diagnosis and treatment of RCC remains a problem worldwide. RCC development is a complicated biological process characterized by a myriad spectrum of molecular abnormalities. Therefore, identification of RCC associated genes and investigation of their clinical significance and functions is of great importance.

Although m^6^A methylation in eukaryotic mRNA has been implicated in diverse biological processes, its mechanisms of action remained unclear [18]. m^6^A methylation is catalyzed by a large multicomponent methyltransferase complex, including three fractions termed METTL3, METTL14 and WTAP [15, 19]. It has been clarified that knockdown of METTL3 could result in apoptosis of human Hela cells and HepG2 cells [16]. Additionally, gene ontology analysis using genes of differential expression demonstrated a remarkably enrichment of p53 signaling pathway [16]. Moreover, studies also showed the depletion of METTL3 homologues in other species may cause developmental arrest or defects in gametogenesis, indicating METTL3 might participate in development and gametogenesis [20, 21]. However, few studies reported METTL3 function in tumorigenesis and development.

In our study, we performed immunohistochemistry in clinical RCC samples microarray to explore METTL3 expression. Based on the results, we analyzed the relationship between METTL3 expression and clinicopathological factors or survival of RCC patients. The results showed that negative METTL3 expression was associated with larger tumor size and higher histological grade. Moreover, RCC patients with positive METTL3 expression had a significantly longer survival time, suggesting METTL3 expression might affect RCC prognosis. Accordingly, we speculated METTL3 might have effects on RCC cell functions such as proliferation, migration and invasion. Consequently, we continued following cell function study with RCC cell lines.

To further explore the function of METTL3 in RCC, we first validated the expression of METTL3 in RCC tissue samples and cell lines, indicating METTL3 expression was significantly lower in RCC samples compared with adjacent non-tumor samples. Moreover, METTL3 was also confirmed lower expressed in RCC cell lines (CAKI-1, CAKI-2 and ACHN) than in HK-2. Subsequently, METTL3 was down-regulated and up-regulated in CAKI-1 and CAKI-2 cell lines, and cell function studies revealed that METTL3 expression was associated with migration, invasion, proliferation and cell cycle of RCC cells. Our data indicated that METTL3 knocked down significantly promoted the abilities of migration, invasion and proliferation of RCC cells. Moreover, METTL3 knockdown decreased G1 arrest, while METTL3 up-regulation promoted G1 arrest. Furthermore, *in vivo* studies were also performed in nude mice using CAKI-1 cell line, and the results were consistent with previous results.

In total, our research clarified the function of METTL3 in RCC tumorigenesis and progression. Moreover, to explore the potential molecular mechanism, our study also revealed that down-regulation of METTL3 could promote the epithelial phenotype and repress a mesenchymal phenotype, while up-regulation of METTL3 could reverse EMT progression. Epithelial cells acquire mesenchymal fibroblast-like properties in the procedure of EMT [22], which may provide cancer cells with motility, invasion and migration functions [23, 24] and contribute to tumor metastatic potential [25, 26]. Moreover, EMT is demonstrated to be associated with prognosis of RCC patients [27]. In our study, the expressions of vimentin, β-catenin and N-cadherin were significantly higher when METTL3 was down-regulated, while the expression of E-cadherin was significantly lower. Such findings were consistent with cell function results, suggesting EMT pathway may be involved in the underlying mechanism.

Furthermore, we also researched the PI3K/Akt/mTOR signaling pathway to explore the underlying molecular mechanism by which METTL3 affects RCC cell proliferation. As reported, the PI3K/AKT/mTOR pathway played a vital role in various cellular processes, such as cell growth, proliferation and survival [28, 29]. In our study, obviously high expressions of p-PI3k, p-AKT, p-mTOR, and p-p70 were observed in METTL3 knockdown group, while such expressions were lower when METTL3 was overexpressed. Moreover, the expression of p-4EBP1 was significantly lower when METTL3 was knocked down. We provided the evidence that METTL3 might affect progression in RCC by affecting PI3K/Akt/mTOR signaling pathway.

In summary, our results showed that higher expression of METTL3 might indicate better survival outcome of RCC patients. Moreover, METTL3 regulated cell proliferation, migration and invasion function in RCC, and EMT and PI3K-Akt-mTOR pathways may be involved in the potential mechanisms. Overall, METTL3 might act as a tumor suppressor in the development, biological progress and survival of RCC patients.

## MATERIALS AND METHODS

### Clinical samples and tissue microarray (TMA) analysis

The study was approved by the institutional review board of the First Affiliated Hospital of Nanjing Medical University. Written informed consent was obtained from all patients included in the study. Clinical samples were collected from RCC patients underwent partial or radical nephrectomy from January 2011 to December 2014 at the Department of Urology of the First Affiliated Hospital of Nanjing Medical University. RCC and matched histologically-normal renal tissue from each case were frozen and stored in liquid nitrogen immediately after resection.The diagnosis of RCC was confirmed by histopathology.

Clinical RCC samples were utilized to make tissue microarray (TMA) which constructed from 145 cases of RCC tumor tissues. TMAs were kept at 4°C until they were ready for analysis. Immunohistochemistry was adopted to explore the expression level of METTL3. Then, the associations of METTL3 expression with clinicopathological features or survival of the RCC patients were analyzed.

### Cell lines, reagents and culture conditions

Human RCC cell lines (CAKI-1, CAKI-2 and ACHN), and a normal renal tubular epithelial cell line (HK-2) were purchased from the Institute of Biochemistry and Cell Biology of the Chinese Academy of Sciences (Shanghai, China). Cells were cultured in McCoy's 5A, RPMI 1640 or DMEM (GIBCO-BRL, Carlsbad, CA, USA) medium supplemented with 10% fetal bovine serum (Gibco/Invitrogen, Australia, Carlsbad, CA, USA),100mg/ml streptomycin and 100U/ml penicillin (Invitrogen, Carlsbad, CA, USA) at 37°C in a humidified incubator with 5% CO_2_.

### Cell transfection

The lentivirus constructs were generated to knockdown and overexpression of METTL3. RCC cells were stably transfected with METTL3 overexpression lentivirus and LV5-EF1a-GFP-Puro negative control vectors, following the manufacturer's instructions (GenePharma, Shanghai, China). For METTL3 knockdown, RCC cells were stably transfected with LV3-pGLV-h1-GFP-puro negative control vectors and METTL3 knockdown lentivirus (Supplementary Table 1). Briefly, cells were plated in 6 wells dishes at 50% confluence and infected with the retroviruses. Meanwhile, polybrene (5 μg/ml) was added with the retroviruses to enhance infection efficiency. Stable pooled populations of RCC cells were generated by selection using puromycin (3 μg/ml) for 2 weeks. For METTL3 knockdown, construct with ≥85% knockdown efficiency was used for further studies.

### RNA extraction and quantitative real-time PCR (qRT-PCR) analyses

Total RNA was extracted from clinical samples or cultured cells with TRIzol reagent (Invitrogen Life Technologies, Carlsbad, CA, USA), according to the manufacturer's protocols. For reverse transcriptase polymerase chain reaction (RT-PCR), RNA reverse transcribed to cDNA from 1μg of total RNA was reverse transcribed in a final volume of 10 μl using random primers and a Reverse Transcription Kit (Perfect Real Time; Takara Biotechnology Co. Ltd., Dalian, China). According to the manufacturer's instructions, the reverse transcription was performed at 37°C for 15 min, then 85°C for 5s and 4°C for ∞. The quantitative real-time PCR (qRT-PCR) were performed on an ABI StepOne Plus instrument (Applied Biosystems, Carlsbad, CA, USA), using a standard protocol from Power SYBR Green (Tli RNaseH Plus; Takara Biotechnology CO. LTD., Dalian, China) according to the manufacturer's instructions and in a total reaction volume of 10 μL, including 5 μL of SYBR Premix (2x), 0.4 μL of PCR forward primer (10 μM), 0.4 μL of PCR reverse primer (10 μM), 0.2 μL ROX Reference Dye II (50x), 1 μL of cDNA, 3 μL of double-distilled water. The qRT-PCR reaction was performed under the following conditions: 95°C for 10 min and 95°C for 30 s, 95°C for 5s, 60°C for 30 s in cycles a total 40 cycles, a final extension step at 72°C for 5 min. mRNA expression in each sample was finally determined after correction with β-actin expression. The gene specific threshold cycle (Ct) for each sample was corrected by subtracting the Ct for the housekeeping gene β-actin (ΔCt). Normal tissues or normal cell line HK-2 were chosen as the control samples, and the ΔCt for all tumor tissues samples or tumor cell lines were subtracted by the ΔCt for the control samples (ΔΔCt). The relative quantitative value of test gene mRNA was expressed as 2^−ΔΔCt^. Each experiment was performed in triplicates and repeated three times.

The PCR primers were as follows: METTL3: Forward: 5’-CAAGCTGCACTTCAGACGAA-3’ Reverse: 5’-GCTTGGCGTGTGGTCTTT-3’ β-actin: Forward: 5’-ACTGGAACGGTGAAGGTGAC-3’ Reverse: 5’-AGAGAAGTGGGGTGGCTTTT -3’.

### Cell proliferation assays

Cell proliferation was assessed by using CCK-8 kit (Dojindo, Kumamoto, Japan) according to the manufacturer's instruction. Briefly, the cells were seeded in 96-well plates at a density of 2×10^3^ cells per well. Duplicate sets of 5 wells each were assessed for each time point. After 24, 48, 72 and 96 h of culture, cells were treated with 10 μL Cell Counting Kit-8 (CCK-8, Dojindo, Kumamoto, Japan) to assess the proliferation potential. The cells were incubated at 37°C for another 2h and then read at 450 nm with a microplate reader (Infinite M200Pro, Tecan, Switzerland). All experiments were performed in triplicate.

### Colony formation assay

For the colony formation assay, cells were trypsinized into single-cell suspension. And 8×10^2^ cells were plated into each media plate and maintained in complete culture medium, replacing the complete culture medium every 4 days. After 2 weeks, colonies were fixed with methanol and stained with 0.1% crystal violet (Beyotime, Beijing, China) respectively for 20 min. Finally, visible colonies were photographed and manually counted. Triplicate plates were counted in each treatment group.

### Cell cycle assay

After treated with trypsin, the cells were collected and washed with PBS twice. Then, the cells were fixed with 70% cold ethanol at -20°C for 2 h, and passed through 70-μm Falcon Filters to obtain a mono-dispersed cell suspension. The mono-dispersed cells were incubated with RNase A and stained with propidium iodide (PI) at room temperature for 30 min (Cell Cycle Detection Kit, BD, USA). Flow-cytometric analysis was performed with a FACS Calibur flow cytometer (Becton Dickinson, Oxford, UK). Finally, the cell cycle would be analyzed utilizing Cell Quest Modfit software.

### Wound-healing migration assay

The cells were seeded into 6-well plates at a density of 1.0×10^5^ cells and cultured in complete medium. A fine pipette tip was used to scratch the confluent monolayer of cells. Then, cells migration into the wound was visualized by microscopy and scored by measuring the size of the initial wound and comparing it to the size of the wound after 48 h.

### Cell migration and invasion assay

Cell migration or invasion assay were performed, respectively using a 24-well transwell chamber (Millicell, PIEP12R48), coated with or without Matrigel (BD Biosciences, Bedford, USA). 2.5×10^4^ cells were suspended in 200 μL with serum free medium, and seeded in the upper chamber of the transwell with a membrane pore with an 8 μm diameter. Culture medium containing 10% FBS was placed in the lower chamber. Cells were incubated for another 48-72 h at 37°C. Cells which passed through the filter were fixed and stained using 0.1% crystal violet. The other cells that did not migrate through the pores of the transwell inserts would be manually removed with a cotton swab. Numbers of invaded cells were counted in five randomly selected fields under a microscope, and the average value was calculated. Each experiment was conducted in triplicate. Matrigel invasion assays were performed as described previously. Each experiment was represented in three individual wells.

### Western blot analysis

Frozen tissues or cells were lysed using the mammalian protein extraction reagent RIPA (Beyotime, Beijing, China) supplemented with a protease inhibitor cocktail (Roche, Shanghai, China) and PMSF (Roche, Shanghai, China) at 4°C for 30 min. Then, cell-debris was removed by centrifugation at 12000 rpm for 10 min. The protein content was measured and determined utilizing the BCA assay (BCA protein assay kit, Pierce Biotechnology, Rockford, IL). The supernatants were mixed with 1/4 volume of 5X sodium dodecyl sulfate sample buffer and boiled for 10 minutes. Protein was separated by 8%-12% sodium dodecyl sulfate–polyacrylamide gel electrophoresis (SDS–PAGE) and then transferred to polyvinylidene difluoride (PVDF) membranes (Bio-Rad, St. Louis, MO, USA). The PVDF membranes were soaked in 5% w/v skim milk at room temperature for 2 h and incubated with the primary antibodies at 4°C overnight. Subsequently, the PVDF membranes were incubated with horseradish peroxidase (HRP)-conjuaged goat anti-rabbit secondary antibodies (Cell Signaling Technology, Danvers, USA) for 1 h at room temperature. Enhanced chemiluminescence (ECL) chromogenic substrate was used to visualize the bands and the intensity of the bands was quantified by Image Lab software (Quantity One software; Bio-Rad, CA, USA). Rabbit monoclonal antibody METTL3, PI3k, p-PI3K, AKT, p-AKT, mTOR, p-mTOR, 4EBP1, p-4EBP1, P70, p-P70, vimentin, β-catenin, N-cadherin and E-cadherin were purchased from Cell Signaling Technology, Inc. (St. Louis, MO, USA). GAPDH was utilized as control.

### Xenograft studies

The study was approved by Medical Laboratory Animal Welfare and Ethics Committee of Nanjing Medical University. BALB/c nude mice were randomly divided into two groups, and each contained four mice. Cells (5×10^6^ cells in 200 μl) were suspended with 100 μl PBS and 100 μl Matrigel Matrix, and injected subcutaneously into the left armpit of each mouse. The volume and weight of the resulting tumors were measured every three days with calipers and electronic scale. The volume was calculated with the formula of length×width^2^×0.52. The mice were humanely sacrificed 3 weeks after injection, and the tumors were dissected. The methods were performed according to the approved guidelines.

### Statistical analysis

All data were presented as mean ± SD (standard deviation) and statistical analysis was performed with SPSS version 17.0 software (IBM, Chicago, IL, USA). Student's t test and chi-square test were used and *P* < 0.05 was considered statistically significant. The Kaplan-Meier method was adopted for survival analysis, and the survival data was conducted with Cox proportional hazards regression model.

## SUPPLEMENTARY MATERIALS TABLES




