# Supplementary material for: The M6A methyltransferase METTL3: acting as a tumor suppressor in renal cell carcinoma
Source: Oncotarget. 2017 Oct 10;8(56):96103–16. doi: 10.18632/oncotarget.21726 (PMC5707084; doi:10.18632/oncotarget.21726)
Supplement: Supplementary file 1 [file oncotarget-08-96103-s001.pdf]

The M6A methyltransferase METTL3: acting as a tumor suppressor in renal cell carcinoma

SUPPLEMENTARY MATERIALS

Supplementary Table 1: 3 selected oligos for sh-METTL3 lentivirus construct

No	Start	Oli Oligo Sequence go Type
Sh-M3-1		
	Top Strand	5'- CACCGCTGCACTTCAGACGAATTATCGAAATAATTCGTCTGAAGTGCAGC -3'
	Bottom Strand	5'- AAAAGCTGCACTTCAGACGAATTATTTCGATAATTCGTCTGAAGTGCAGC -3'
	ds Oligo	5'- CACCGCTGCACTTCAGACGAATTATCGAAATAATTCGTCTGAAGTGCAGC -3'
		| | | | | | | | | | | | | | | | | | | | | | | | | | | | | | | | | | | | | | | | | | | | | |
		3'- CGACGTGAAGTCTGCTTAATAGCTTTATTAAGCAGACTTCACGTCGAAAA -5'
Sh-M3-2		
	Top Strand	5'- CACCGGATACCTGCAAGTATGTTCACGAATGAACATACTTGCAGGTATCC -3'
	Bottom Strand	5'- AAAAGGATACCTGCAAGTATGTTCATTCGTGAACATACTTGCAGGTATCC -3'
	ds Oligo	5'- CACCGGATACCTGCAAGTATGTTCACGAATGAACATACTTGCAGGTATCC -3'
		| | | | | | | | | | | | | | | | | | | | | | | | | | | | | | | | | | | | | | | | | | | | | |
		3'- CCTATGGACGTTCATACAAGTGCTTACTTGTATGAACGTCCATAGGAAAA -5'
Sh-M3-3		
	Top Strand	5'- CACCGCTCAACATACCCGTACTACACGAATGTAGTACGGGTATGTTGAGC -3'
	Bottom Strand	5'- AAAAGCTCAACATACCCGTACTACATTCGTGTAGTACGGGTATGTTGAGC -3'
	ds Oligo	5'- CACCGCTCAACATACCCGTACTACACGAATGTAGTACGGGTATGTTGAGC -3'
		| | | | | | | | | | | | | | | | | | | | | | | | | | | | | | | | | | | | | | | | | | | | | |
		3'- CGAGTTGTATGGGCATGATGTGCTTACATCATGCCCATACAACTCGAAAA -5'

Mettl3 : XM_006720206.1 http://www.ncbi.nlm.nih.gov/nuccore/XM_006720206.1 (The enzyme loci were marked by yellow).

GCTAGCATGAAAGCCAGTGATGCTGATCGACCCTGTCGCAAGCTGCACTTCAGACGAATTATCAATAAACACA
CTG.
ATGAGTCTTTAGGTGACTGCTCTTTCCTTAATACATGTTTCCACATGGATACCTGCAAGTATGTTCACTA
TGAAATTGATGCTTGCATGGATTCTGAGGCCCCTGGCAGCAAAGACCACACGCCAAGCCAGGAGCTTGCT
CTTACACAGAGTGTCGGAGGTGATTCCAGTGCAGACCGACTCTTCCCACCTCAGTGGATCTGTTGTGATA
TCCGCTACCTGGACGTCAGTATCTTGGGCAAGTTTGCAGTTGTGATGGCTGACCCACCCTGGGATATTCA
CATGGAACTGCCCTATGGGACCCTGACAGATGATGAGATGCGCAGGCTCAACATACCCGTACTACAGGAT
GATGGCTTTCTCTTCCTCTGGGTCACAGGCAGGGCCATGGAGTTGGGGAGAGAATGTCTAAACCTCTGGG
GGTATGAACGGGTAGATGAAATTATTTGGGTGAAGACAAATCAACTGCAACGCATCATTCGGACAGGCCG
TACAGGTCACTGGTTGAACCATGGGAAGGAACACTGCTTGGTTGGTGTCAAAGGAAATCCCCAAGGCTTC
AACCAGGGTCTGGATTGTGATGTGATCGTAGCTGAGGTTCGTTCCACCAGTCATAAACCAGATGAAATCT
ATGGCATGATTGAAAGACTATCTCCTGGCACTCGCAAGATTGAGTTATTTGGACGACCACACAATGTGCA
ACCCAACTGGATCACCCTTGGAAACCAACTGGATGGGATCCACCTACTAGACCCAGATGTGGTTGCACGG
TTCAAGCAAAGGTACCCAGATGGTATCATCTCTAAACCTAAGAATTTATAGGGCGCGCC.

Supplementary Table 2: Characteristics of the 145 RCC patients involved in this study

See Supplementary File 1
