# Supplementary material for: The M6A methyltransferase METTL3: acting as a tumor suppressor in renal cell carcinoma
Source: Oncotarget. 2017 Oct 10;8(56):96103–16. doi: 10.18632/oncotarget.21726 (PMC5707084; doi:10.18632/oncotarget.21726)
Supplement: Supplementary file 2 [file oncotarget-08-96103-s002.doc]

Supplements 2. Characteristics of the 145 RCC patients involved in this study.

| Age |  |  |
| --- | --- | --- |
| Mean±SD, year | 55.13±14.16 |  |
| ＜50 | 52 | 35.9% |
| 50-60 | 36 | 24.8% |
| ≥60 | 57 | 39.3% |
| Gender |  |  |
| Male | 89 | 61.4% |
| Female | 56 | 38.6% |
| Smoking status |  |  |
| Never | 91 | 62.8% |
| Ever | 54 | 37.2% |
| Drinking status |  |  |
| Never | 106 | 73.1% |
| Ever | 39 | 26.9% |
| Hypertension |  |  |
| Never | 83 | 57.2% |
| Ever | 62 | 42.8% |
| Diabetes |  |  |
| Never | 118 | 81.4% |
| Ever | 27 | 18.6% |
| Cancer of kidney |  |  |
| Left | 78 | 53.8% |
| Right | 65 | 44.8% |
| Both | 2 | 1.4% |
| Tumor size |  |  |
| Mean±SD, cm | 4.85±2.55 |  |
| ≤4 | 72 | 49.7% |
| ＞4 | 73 | 50.3% |
| Histology |  |  |
| Clear cell carcinoma | 127 | 87.6% |
| Others | 18 | 12.4% |
| Histological grade |  |  |
| Ⅰ | 38 | 26.2% |
| Ⅱ | 66 | 45.5% |
| Ⅲ | 29 | 20.0% |
| Ⅳ | 12 | 8.3% |
| Tumor stage |  |  |
| T1 | 106 | 73.1% |
| T2 | 21 | 14.5% |
| T3 | 11 | 7.6% |
| T4 | 7 | 4.8% |
| Survival |  |  |
| Mean±SD, month | 62.32±26.57 |  |
| No | 42 | 29.0% |
| Yes | 103 | 71.0% |
| METTL3 expression |  |  |
| Negative | 117 | 80.7% |
| Positive | 28 | 19.3 |

SD, standard deviation
